# Active Flavonoids From *Lagotis brachystachya* Attenuate Monosodium Urate-Induced Gouty Arthritis *via* Inhibiting TLR4/MyD88/NF-κB Pathway and NLRP3 Expression

**DOI:** 10.3389/fphar.2021.760331

**Published:** 2021-11-05

**Authors:** Xiang Ouyang, Na-Zhi Li, Min-Xia Guo, Man-Man Zhang, Jie Cheng, Li-Tao Yi, Ji-Xiao Zhu

**Affiliations:** ^1^ Research Center of Natural Resources of Chinese Medicinal Materials and Ethnic Medicine, Jiangxi University of Chinese Medicine, Nanchang, China; ^2^ Department of Chemical and Pharmaceutical Engineering, Huaqiao University, Xiamen, China

**Keywords:** *Lagotis brachystachya*, NF-κB, NLRP3, gouty arthritis, monosodium urate

## Abstract

*Lagotis brachystachya* Maxim is a characteristic herb commonly used in Tibetan medicine. Tibetan medicine records it as an important medicine for the clinical treatment of “Yellow Water Disease,” the symptoms of which are similar to that of arthritis. Our previous study showed that the flavonoid fraction extracted from *L. brachystachya* could attenuate hyperuricemia. However, the effects of the active flavonoids on gouty arthritis remain elusive, and the underlying mechanism is not understood. In the present study, the effects of the active flavonoids were evaluated in rats or Raw264.7 cells with gouty arthritis induced by monosodium urate (MSU) crystal, followed by the detection of TLR4, MyD88, pNF-κB, and NLR family pyrin domain-containing 3 (NLRP3) expression. The swelling of the ankle joint induced by MSU crystal began to be relieved 6 h post the administration with the active flavonoids. In addition, the active flavonoids not only alleviated MSU crystal-induced inflammation in synovial tissues by histopathological examination but also reduced tumor necrosis factor alpha (TNF-α) and interleukin-1 beta (IL-1β) levels in the joint tissue fluid of MSU crystal-induced rats. Furthermore, Western blot analysis indicated that the active flavonoids reduced the production of these cytokines by inhibiting the TLR4/MyD88/NF-κB pathway and decreasing NLRP3 expression in synovial tissues of rats. More importantly, the inhibition of TLR4/MyD88/NF-κB pathway and NLRP3 expression was also confirmed in MSU-induced Raw264.7 cells. In conclusion, these results indicated that the active flavonoids from *L. brachystachya* could effectively attenuate gouty arthritis induced by MSU crystal through the TLR4/MyD88/NF-κB pathway and NLRP3 expression *in vivo* and *in vitro*, suggesting several potential candidates for the treatment of gouty arthritis.

## 1 Introduction

Gouty arthritis is characterized by the deposition of urate in the joint capsule, bursa, cartilage, bone, and other tissues, accompanied by inflammation (Ragab et al., 2017). It is reported that the main pathogenesis of gouty arthritis is the disorder of the purine metabolism of the body’s, which leads to a high concentration of uric acid in the blood and the precipitation of sodium urate crystal and deposits in the joints, which activates the inflammatory response (Ghaemi-Oskouie and Shi, 2011).

Monosodium urate crystal is the causative factor of gout, which can activate a variety of immune cells to release proinflammatory cytokines such as tumor necrosis factor-alpha (TNF-α) and interleukin-1 beta (IL-1β), thereby inducing the production of gouty arthritis (Desai et al., 2017). The release of proinflammatory cytokines is regulated by a variety of signaling pathways after MSU crystal injection, among which the TLRs/MyD88/NF-κB (Chen et al., 2006) and NLR family pyrin domain-containing 3 (NLRP3) (Amaral et al., 2012) signaling pathways are the main regulatory signaling pathways. The two inflammatory signaling pathways can act independently but also can coordinately regulate the key factors released by inflammation.

*Lagotis brachystachya* Maxim, a traditional Tibetan medicine, is recorded as an important medicine for the clinical treatment of “Yellow Water Disease.” The symptoms of Yellow Water Disease are similar to that of arthritis in traditional Chinese Medicine (BianBa et al., 2019). Therefore, it is primarily used to alleviate inflammation-related disease such as gout, gouty arthritis in the local Tibet of China, for a long time (Luo, 2004). However, the detailed mechanism underlying its efficacy remains unknown. In our previous study, the flavonoid fraction extracted from *L. brachystachya* could attenuate hyperuricemia in mice (Xiong et al., 2018). In addition, three active flavonoids including luteolin, luteoloside, and apigenin were isolated from *Lagotis brachystachy* (Zhu et al., 2019). According to the previous reports, the active flavonoids luteolin, luteoloside, and apigenin have been shown to exert anti-inflammatory activity *in vivo* (Nishitani et al., 2013; Luan et al., 2016; Li et al., 2019). More importantly, a recent study indicated that luteolin-attenuated MSU crystal induced- gouty arthritis via inhibiting the TLR/MyD88/NF-κB pathway (Shen et al., 2020). Similarly, luteolin-4′-O-glucoside, a structural analog of luteoloside was shown to alleviate paw swelling via decreasing serum pro-inflammatory cytokine in MSU crystal-induced gouty arthritis (Lin et al., 2018). Meanwhile, several previous studies have shown that luteolin (Dong et al., 2021; Lee et al., 2021), luteoloside (Fan et al., 2014; Wang, Z. et al., 2021), and apigenin (Zhang et al., 2014; Zhao et al., 2019) could inhibit TLR4 signaling and NLRP3 expression. Therefore, the putative effects of luteolin, luteoloside, and apigenin on TLR4 and NLRP3 were first evaluated by molecular docking in our previous study (Zhu et al., 2021). The results showed that luteolin, luteoloside, and apigenin could enter into the inhibitory pockets of TLR4 and NLRP3, indicating the potential roles in the inhibition of inflammation. These observations above provided a speculation that these three flavonoids might alleviate gouty arthritis *via* the TLR4/MyD88/NF-κB pathway and NLRP3 expression. Therefore, the effects and underlying mechanism of the three active flavonoids (luteolin, luteoloside, and apigenin) were investigated *in vivo* and *in vitro*.

## 2 Materials and Methods

### 2.1 Animals

Male Kunming rats (200 ± 20 g) were purchased from the Animal Center of Jiangxi University of Chinese Medicine, China. The animals were housed individually in a cage (320 × 180 × 160 cm) under a normal 12 h/12 h light/dark schedule (lights on at 07:00 am) during the experiments. The animals were allowed 1 week to adapt prior to the formal experiments. Ambient temperature and relative humidity were maintained at 22 ± 2°C and 55 ± 5%. Food and water were freely available to the animals. The animal experiments complied with the ARRIVE guidelines and were approved by the Jiangxi University of Chinese Medicine (JZLLSC2019-0221). All procedures were performed following the published guidelines of the China Council on Animal Care.

### 2.2 Reagents

Uric acid (batch number BCBH0278V) was purchased from Sigma-Aldrich Co., Ltd. (St. Louis, MO, United States). Colchicine (batch number 20190901) was purchased from Xishuangbanna Pharmaceutical Co., Ltd. Rat IL-1β (batch number MM0047R1) and TNF-α (batch number MM0180R1) ELISA kits were purchased from Meimian Co., Ltd. (Yancheng, China). Mouse TNF-α (batch number CSB-E04741m) ELISA kit was purchased from Cusabio Co., Ltd. (Wuhan, China). Primary antibodies against TLR2 (ab213676), TLR4 (ab217274), MyD88 (ab2064), NF-κB (ab76302), NLRP3 (ab214185), caspase-1 (ab138483), and IL-1β (ab9772) were purchased from Abcam Inc. (Cambridge, MA, United States). RAW264.7 cells were purchased from the Cell Bank of the Chinese Academy of Sciences (Shanghai, China).

### 2.3 Isolation of Luteolin, Luteoloside, and Apigenin from *Lagotis brachystachya*

*Lagotis brachystachya* Maxim, collected from the Sichuan Province of China in 2015 was identified by Professor Guo-Yue Zhong (Jiangxi University of Chinese Medicine). A voucher specimen (No. 01-03-23-15) was deposited at the research center. Luteolin, luteoloside, and apigenin were isolated according to our previous study (Zhu et al., 2019).

### 2.4 Preparation of Monosodium Urate Crystal

The method for MSU crystal preparation was according to the previous study (Yin et al., 2020). Briefly, uric acid was added first to NaOH solution for boiling to be dissolved. Then the solution was cooled down, and the pH was adjusted to 8.9 with NaOH solution, followed by staying at 4°C overnight. The supernatant was removed the next day, and the residue was dried at 60°C for 2 h. Finally, the MSU crystal powder was obtained after high-temperature sterilization.

### 2.5 Drug treatment *In Vivo*

**FIGURE 1:**
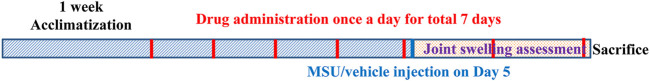
Timeline of the present study.

Gouty arthritis is initiated by the deposition of MSU crystal around the joints. Therefore, the acute gouty arthritis model was widely established by injection of MSU crystal into the ankle joint of rodents (Pineda et al., 2015). The rats were randomly divided into the following groups (n = 9): control-vehicle group, MSU-vehicle (Model) group, MSU-colchicine group (10 mg/kg), MSU-luteolin group (30, 60 mg/kg), MSU-luteoloside (30, 60 mg/kg), and MSU-apigenin (30, 60 mg/kg). Rats were orally administrated for seven continuous days. On the fifth day, MSU crystal (5 mg of dissolved in saline) was injected into the right posterior ankle of rats 1 h after drug administration. On the seventh day, the animals were anesthetized with 10% chloral hydrate and sacrificed 1 h post drug administration. The synovial tissues of right posterior ankle were dissected for histopathological examination and Western blot. The timeline of the present study is shown in Figure 1.

The doses (30 and 60 mg/kg) of the active flavonoids were selected according to our preliminary experiment.

### 2.6 Joint Swelling Assessment

The joint swelling was measured at the same position 4, 6, 8, 12, 16, 24, and 48 h after MSU crystal injection. The degree of swelling was calculated by the following formula: circumference of ankle joint after MSU crystal injection—circumference of ankle joint before MSU crystal injection)/circumference of ankle joint before MSU crystal * 100%.

### 2.7 Drug Treatment *In Vitro*

RAW264.7 cells were purchased from Cell Bank of the Chinese Academy of Sciences (Shanghai, China) and cultured in DMEM complete medium. The third generation of RAW264.7 cells were used to incubate in a 96-well plate (5.0 × 10^3^/ml for each well) and were randomly divided into control-vehicle group, MSU-vehicle (Model) group, MSU-colchicine group (0.001 μmol/L), MSU-luteolin groups (3.125, 6.25 μmol/L), MSU-luteoloside (3.125, 6.25 μmol/L), and MSU-apigenin (3.125, 6.25 μmol/L). The RAW264.7 cells were pretreated with different concentrations of active flavonoids mentioned above for 1 h and then stimulated with 0.0625 mg/ml of MSU for 12  h, followed by biochemical analysis. In our preliminary study, the concentration gradients of MSU crystal (0.015625, 0.03125, 0.0625, 0.125, 0.25, 0.5, 1 mg/ml) were used for different times (6, 12, 24 h). We found that 0.0625 mg/ml treated for 12 h was the optimal concentration to activate IL-1β release.

### 2.8 Enzyme-Linked Immunosorbent Assay Analysis

Saline was injected into the joint cavity. After moderate bending, extension, and rotation, the mixed synovial fluid of the hind limbs was mixed with saline, and the joint tissue fluid was extracted from the outside of the trigeminal puncture. The joint tissue fluid was collected and centrifuged at 300 ×*g* at 4°C for 10 min followed by collecting the supernatant. Then the concentrations of proinflammatory cytokines (TNF-α and IL-1β) were evaluated by ELISA kits according to the instruction.

### 2.9 Histopathological Examination

After fixing with 4% paraformaldehyde, the synovial tissues were cut into pieces. The cut tissues were rinsed with water for 2 h followed by dehydration with 50, 70, 80, 90%, and absolute ethanol. Then the tissue was dehydrated with xylene and paraffin. After embedding in paraffin, 4 μm slices were cut and collected. Next, the slices were dewaxed with xylene, ethanol, and distilled water. Subsequently, the slices were placed in hematoxylin and eosin (H&E) staining solution. Finally, the slices were sealed and then observed under a microscope. The histological examination was assessed by an observer blind to the treatment. The score of inflammation around was judged by H&E staining from four rats per group, and the standards were as follows: 0, no inflammatory cells; 1, a few inflammatory cells; 2, more unevenly distributed inflammatory cells with local inflammation infiltration; 3, many uniformly distributed inflammatory cells, rarely clustered together; 4, many inflammatory cells aggregate into clusters. The average of three replicates per rat was used for statistical analysis.

### 2.10 Western Blot

The synovial tissues were first homogenized in lysis buffer (including 20 mM Tris (pH7.5), 150 mM NaCl, 1% Triton X-100, and sodium pyrophosphate, β-glycerophosphate, EDTA, Na_3_VO_4_, protease, and phosphatase inhibitors) by a glass homogenizer. Then the homogenates were incubated in a shaker at 4°C for 30 min to further extract proteins. The homogenates were centrifuged at 12,000 ×*g* at 4°C for 15 min. Then the supernatant was retained and adjusted to the same protein concentrations. An equal amount of protein (30 μg in 10 μl) was loaded and separated in the SDS-PAGE system. Then the proteins in the gel were transferred to a PVDF membrane followed by blocking with 5% BSA. Later, the membrane was incubated with antibodies at 4°C overnight (TLR2, 1:2,000; TLR4, 1:2,000; MyD88, 1:2,000; pNF-κB, 1:2,000; NLRP3, 1:2,000; caspase-1, 1:2,000; IL-1β, 1:2,000; β-actin, 1:4,000). On the second day, the membrane was incubated with an HRP-conjugated antibody (1:2,000) at room temperature for 1 h. Finally, the membrane was exposed with enhanced chemiluminescence solution. The gray value of the bands was analyzed by ImageJ software.

### 2.11 Statistical Analyses

The data were expressed as means ± SEM. First of all, the normal distribution of the data was assessed by the Kolmogorov–Smirnov test. Then the data were analyzed by one-way ANOVA followed by Tukey’s *post-hoc* test for multiple comparisons between any two treatment groups. A value of *p *< 0.05 was considered as a significant difference.

## 3 Results

### 3.1 Active Flavonoids Attenuated Gouty Arthritis Related Symptoms in Rats Induced by Monosodium Urate Crystal

**FIGURE 2:**
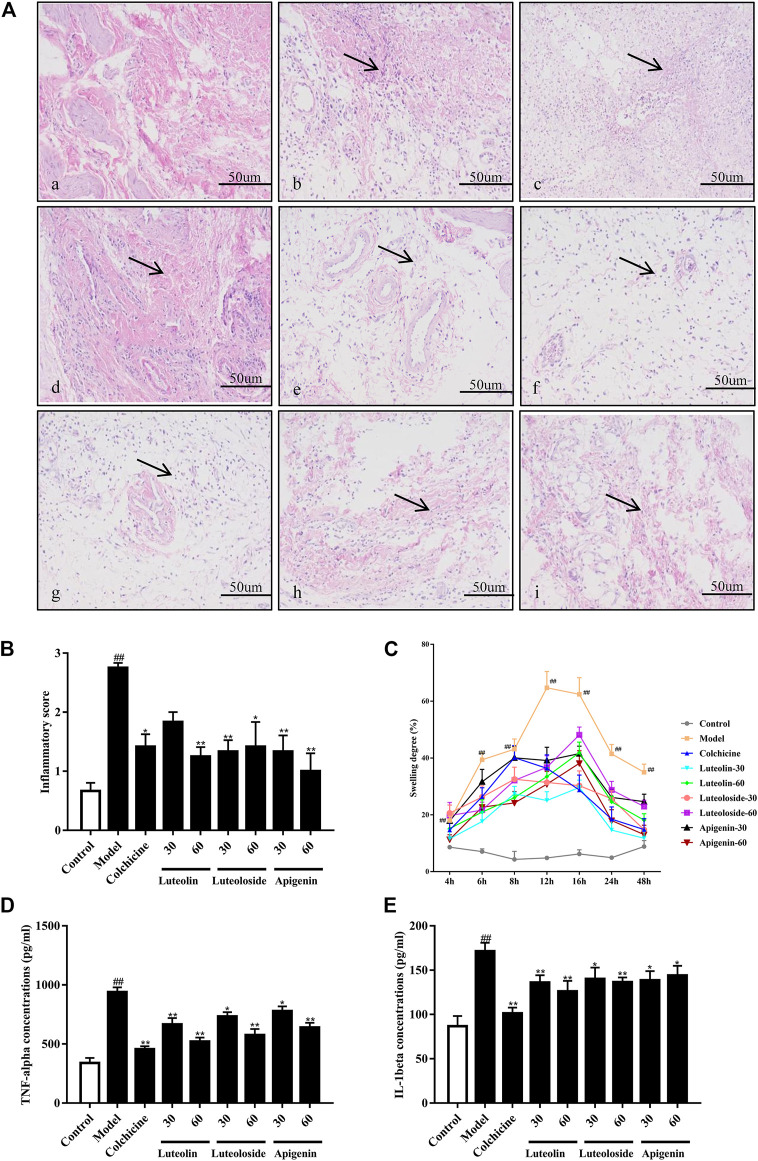
The effects of the active flavonoids luteolin, luteoloside, and apigenin attenuated monosodium urate (MSU) crystal-induced gouty arthritis of rats. Representative images of hematoxylin and eosin (H&E) staining **(A)** of synovial tissue 48 h post injection in **(a)** control-vehicle; **(b)** MSU-vehicle (Model); **(c)** MSU-colchicine; **(d)** MSU-luteolin at 30 mg/kg; **(e)** MSU-luteolin at 60 mg/kg; **(f)** MSU-luteoloside at 30 mg/kg; **(g)** MSU-luteoloside at 60 mg/kg; **(h)** MSU-apigenin at 30 mg/kg; **(i)** MSU-apigenin at 60 mg/kg. The black arrow indicates the inflammatory infiltration in synovial tissue. Scale bar was equal to 50 μm. **(B)** The inflammatory score of synovial tissue based on H&E staining (*n* = 4). **(C)** The joint swelling at different time points. **(D)** Serum tumor necrosis factor alpha (TNF-α) levels. **(E)** Serum interleukin-1 beta (IL-1β) levels.

As shown in Figure 2A, the cells in the synovial tissue of the normal group were normal without infiltration. However, the synovial tissue of the model group showed severe inflammatory cell infiltration, and the surrounding cells show a vesicular-like morphology. The administration with colchicine and active flavonoids can significantly reverse the inflammatory pathological changes of the synovial tissue in the ankle joint. As shown in Figure 2B, the active flavonoids reduced the cell infiltration induced by MSU crystal according to the inflammatory score, the quantified results of histopathological examination.

As shown in Figure 2C, compared with the control-vehicle group, the ankle joint of the MSU crystal-induced rats showed obvious swelling at each time point ranging from 4 to 48 h after MSU crystal injection. The peak of swelling reached 12 h post MSU crystal injection. Subsequently, the swelling gradually decreased within 12–24 h, but it was still significantly higher than that in control rats. Compared with the model group, colchicine and active flavonoids can significantly reduce the swelling of the ankle joint from 6–48 h post MSU crystal injection.

As shown in Figures 2D,E, the MSU crystal significantly increased TNF-α and IL-1β levels in the serum of rats. However, compared with the model group, both colchicine and active flavonoids significantly reduced the levels of TNF-α and IL-1β in response to MSU crystal injection.

### 3.2 The Active Flavonoids Inhibited Synovial TLR4/MyD88/NF-κB and NLRP3 Levels in Gouty Arthritis Rats Induced by Monosodium Urate Crystal

**FIGURE 3:**
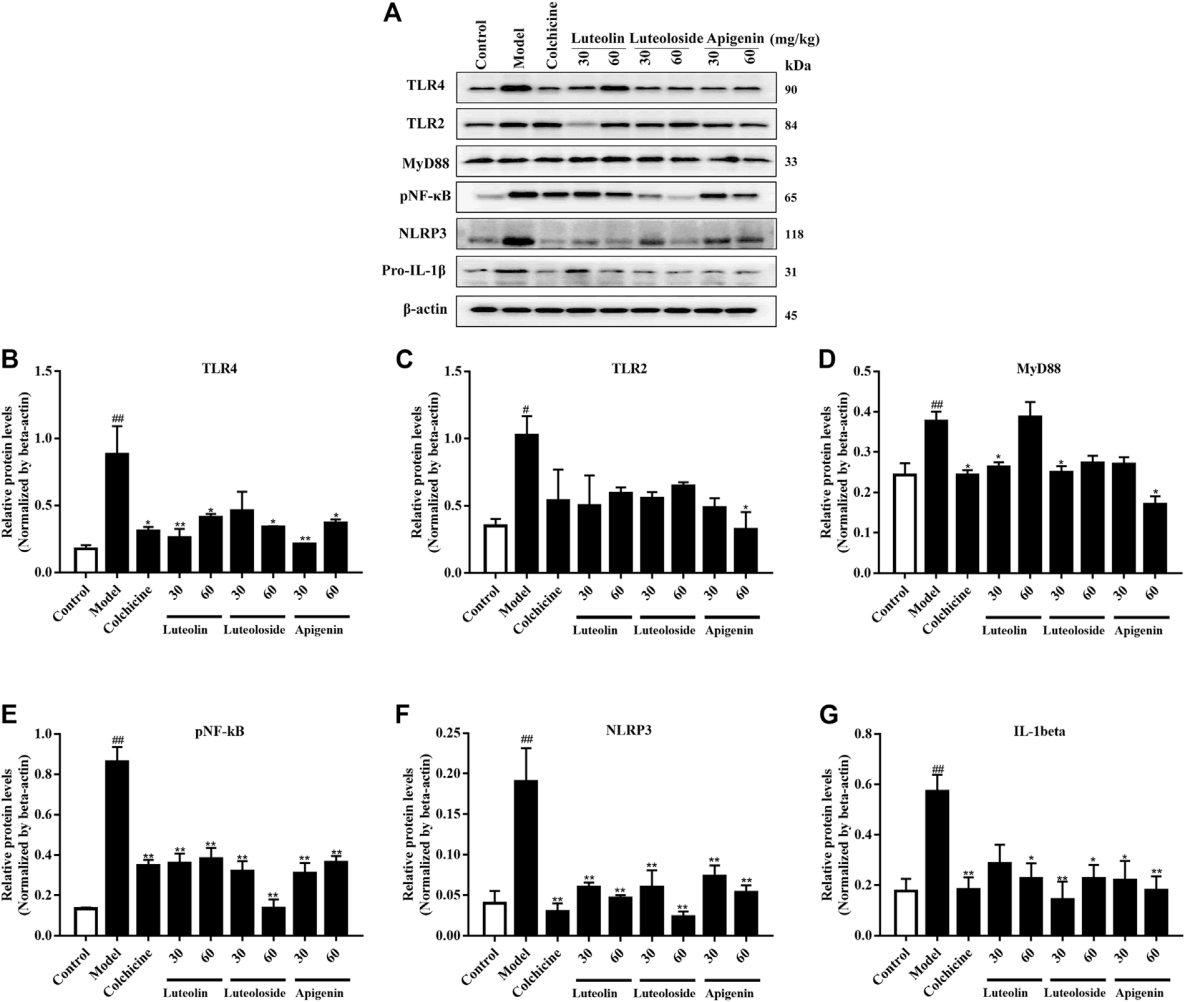
The effects of the active flavonoids luteolin, luteoloside, and apigenin on the TLRs/MyD88/NF-κB pathway and NLRP3/IL-1β expression in MSU crystal-induced gouty arthritis of rats (*n* = 5). The representative western bands are shown in panel **(A)**. The changes in TLR4 **(B)**, TLR2 **(C)**, MyD88 **(D)**, pNF-κB **(E)**, NLRP3 **(F)**, and pro-IL-1β **(G**) are shown as histograms. ^#^ *p* < 0.05 and ^##^ *p* < 0.01 vs. control group; *p < 0.05 and **p < 0.01 vs. model group.

As shown in Figure 3, the levels of TLR2, TLR4, MyD88, and pNF-κB were significantly enhanced by MSU crystal injection, suggesting the upregulation of inflammatory-related pathway in the synovial tissues. Similarly, the NLRP3 levels were also increased by MSU crystal. On the contrary, both colchicine and active flavonoids inhibited TLR4/MyD88/NF-κB as well as NLRP3 levels in the synovial tissues. However, the active flavonoids did not exert a significant change in TLR2 levels in the synovial tissues.

### 3.3 The Active Flavonoids Inhibited TLR4/MyD88/NF-κB Pathway and NLRP3/Caspase-1/IL-1β Expression in Raw264.7 Cells

**FIGURE 4:**
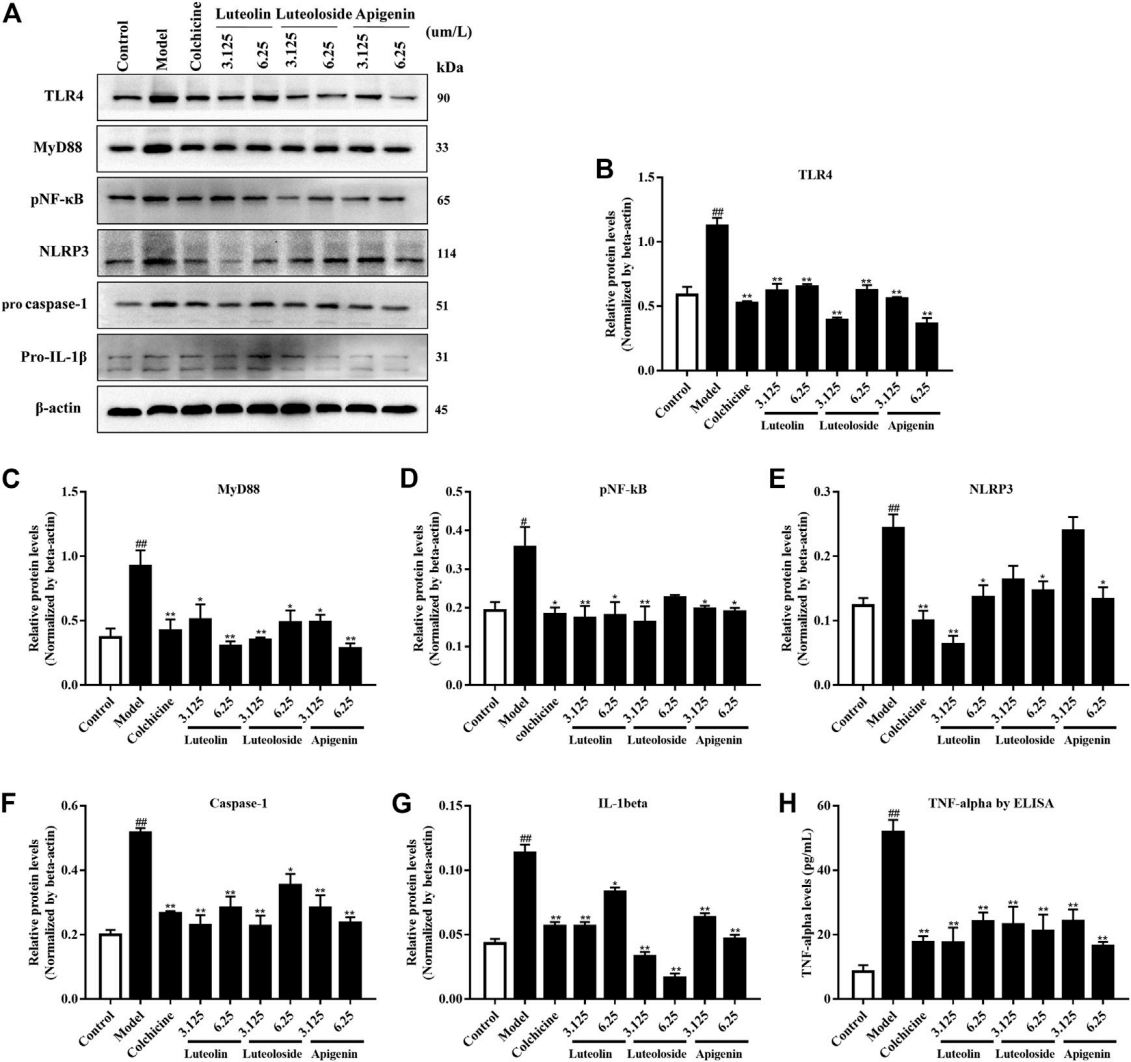
The effects of the active flavonoids luteolin, luteoloside, and apigenin on the TLRs/MyD88/NF-κB pathway, NLRP3/caspase-1/IL-1β expression, and TNF-α levels in MSU crystal-induced Raw264.7 cells (*n* = 4). The representative western bands are shown in panel **(A)**. The changes in TLR4 **(B)**, MyD88 **(C)**, pNF-κB **(D)**, NLR family pyrin domain-containing 3 (NLRP3) **(E)**, pro caspase-1 **(F)**, and pro-IL-1β **(G)** are shown as histograms. **(H)** TNF-α levels by ELISA assay. ^#^ *p* < 0.05 and ^##^ *p* < 0.01 vs. control group; *p < 0.05 and **p < 0.01 vs. model group.

In addition to the *in vivo* experiment, the effects of the active flavonoids were evaluated *in vitro*. As shown in Figure 4, the MSU crystal induced the activation of the TLR4/MyD88/NF-κB pathway and NLRP3/caspase-1/IL-1β expression in Raw 264.7 cells. Compared with the model group, the active flavonoids luteolin, luteoloside, and apigenin can significantly reduce TLR4, MyD88, pNF-κB, caspase-1, and IL-1β levels. Only the high dose of active flavonoids inhibited NLRP3 levels in response to MSU crystal treatment. Furthermore, all the active flavonoids decreased the TNF-α level in MSU crystal-induced Raw264.7 cells.

## 4 Discussion

It is well known that MSU crystal deposition is the clinicopathological basis of gouty arthritis (Dalbeth et al., 2019). In this study, acute gouty arthritis rats were constructed by MSU crystal injection to investigate the therapeutic effects of the active flavonoids extracted from *Lagotis brachystachya*. Exogenous MSU crystal injection can cause acute inflammation in the knee joint of rats, resulting in inflammatory exudation of the synovium, congestion, and swelling of the tissues around the joints, causing joint swelling and increasing circumference, and the joint swelling thus increases (Pineda et al., 2015). In this way, joint swelling, which can reflect the degree of improvement on acute joint inflammation, is widely used to evaluate the anti-gouty arthritis effect of treatment (Lin et al., 2021). In the present study, the swelling of the ankle joint began to be relieved 6 h post the administration with active flavonoids. The positive colchicine also decreased the swelling of the ankle joint, which was in accordance to a recent study (Fu et al., 2021). Moreover, this recovery lasted without intermission to the end of the measurement. These data indicated that the active flavonoids possessed a therapeutic effect against gouty arthritis.

Gouty arthritis has been shown to activate inflammatory cytokines in the body during an acute attack (Jeong et al., 2019). In the early stage of inflammation, the local temperature of the joint is normal. With the increase in uric acid levels in the blood, the urate crystal, initially deposited in the connective tissue near the joint, fluctuates and falls off. When the adhesion of urate crystal reaches a certain critical value, they begin to enter the joint cavity and diffuse. At the same time, the inflammatory response control mechanism of the body is activated with a combination of urate crystal and immunoglobulin (McWherter et al., 2018), thereby causing local inflammation and increase the aggregation of phagocytes (Wu et al., 2020). Accumulating studies have shown that TNF-α and IL-1β are closely related to the pathogenesis of gouty arthritis (Mitroulis et al., 2013; Dinarello and Joosten, 2016). Thus, these two proinflammatory cytokines play a crucial role in the occurrence and relief of gouty arthritis (Pamuk et al., 2008; Dinarello, 2010). More importantly, TNF-α and IL-1β are not only the initiating factors of the inflammatory response but also the continuous inducing factors of inflammation (Joosten et al., 2016; Joers et al., 2020; Sluiter et al., 2020). For example, TNF-α could potentiate uric acid-induced IL-1β secretion and release (Yokose et al., 2018). In contrast, IL-1 and TNF receptor antagonists could significantly relieve the symptoms of patients with gout (Terkeltaub et al., 2009; Hasikova et al., 2019). In the present study, ELISA assay showed that the active flavonoids could significantly decrease the levels of proinflammatory cytokines, TNF-α and IL-1β, in the joint tissue fluid of MSU crystal-induced rats, indicating that luteolin, luteoloside, and apigenin participate in the regulation of inflammatory factors, which mediates the pathophysiology of gouty arthritis in MSU crystal-induced rats. Consistently, two previous studies presented similar results showing that luteolin and luteolin-4′-O-glucoside, a structural analog of luteoloside, decreased serum TNF-α and IL-1β concentrations in MSU crystal-induced gouty arthritis (Lin et al., 2018; Shen et al., 2020).

Next, the histopathological examination confirmed the inflammation of gouty arthritis induced by MSU crystal, which was in accordance with the previous studies showing a similar inflammatory infiltration (Caution et al., 2019; Han et al., 2021). In contrast, the local inflammatory cell infiltration in the joint synovial tissue of MSU crystal-induced rats was reduced by administration with the active flavonoids. The pathological changes in the tissues suggested that the active flavonoids were effective against inflammation-related symptoms.

In the stage of acute gouty arthritis, the MSU crystal activates TLRs such as TLR2 and TLR4 (Qing et al., 2014). TLRs are a type I transmembrane protein, and they can bind to MyD88 and recruit interleukin 1 receptor-associated kinase (IRAK). NF-κB is finally activated under the catalysis of IKB kinase (Brown et al., 2011). Activated NF-κB enters the nucleus and switches on the transcription of inflammatory factors (Kawasaki and Kawai, 2014). Therefore, inhibiting the chain reaction of inflammation has become a target for alleviating the symptoms of gouty arthritis. In the present study, the results indicated that the MSU crystal increased TLR4, MyD88, and pNF-κB, while the active flavonoids reversed the elevation. Our present results were partly in accordance with a previous study indicating that luteolin inhibited the TLR/MyD88/NF-κB pathway, thereby attenuating MSU crystal-induced gouty arthritis (Shen et al., 2020). On the other hand, except for apigenin at 60 mg/kg, the active flavonoids did not reverse the overexpression of TLR2 induced by the MSU crystal, indicating that luteolin and luteoloside mainly regulate the TLR4-dependent pathway.

NLRP3 is a member of NOD-like receptor proteins. As an endogenous danger signal, the MSU crystal can be recognized by NOD-like receptors, activate NLRP3 inflammasome, and produce IL-1β (Jhang et al., 2015). Briefly, the maturation of IL-1β needs to go through the following two stages (Ismael et al., 2021): The first stage is that the MSU crystal acts on cell surface TLRs to activate NF-κB, which causes IL-1β and NLRP3 gene transcription and subsequently generates inactive IL-1β precursors. In the second stage, NLRP3 inflammasome, which is activated by the MSU crystal via different pathways, will activate caspase-1 and, thus, cleave IL-1β precursors into mature IL-1β (Kelley et al., 2019; Yi et al., 2021). If there is only the activated TLRs signaling pathway but not the activation of NLRP3, the precursor IL-1β cannot exert any biological activity. In this context, TLRs and NLRP3 inflammasome cooperate in the process of IL-1β production and activation. The NLRP3 expression was significantly activated in the present study, indicating that the MSU crystal activated the NLRP3 inflammasome. The three active flavonoids could significantly reduce NLRP3 expression, indicating that luteolin, luteoloside, and apigenin might inhibit the activation of NLRP3 inflammasomes, thereby inhibiting the production of IL-1β. There were several publications that indicated that luteolin, luteoloside, and apigenin inhibited NLRP3 activation in rodents (Fan et al., 2014; Lv et al., 2019; Wang, X. et al., 2021); however, to the best of our knowledge, the present study is the first research that investigates the involvement of NLRP3 inhibition in the anti-gouty arthritis effects of luteolin, luteoloside, and apigenin. When NLRP3 inflammasome was activated by damage-associated molecular patterns (DAMPs) or pathogen-associated molecular pattern molecules (PAMPs), it undergoes oligomerization to the adaptor ASC followed by caspase-1 recruiting. Then inflammatory factors are activated by cleavage from inactive precursor forms. Therefore, targeted inhibition of the activation of NLRP3/ASC/caspase-1 can reduce the inflammatory response (Jhang et al., 2018). However, this presents only detected NLRP3 levels but not ASC or caspase-1 levels. In this respect, the present study did not provide a precise regulatory mechanism of NLRP3/ASC/Caspase-1 for the active flavonoids. This is one of the limitations in the present study.

Finally, MSU crystal-induced Raw264.7 cells were used to confirm the mechanism of the active flavonoids. The results of cell experiments were highly consistent with the results of the animal experiments, further suggesting that the active flavonoids can attenuate gouty arthritis by regulating the TLR4/MyD88/NF-κB pathway and NLRP3 levels. NLRP3 inflammasome inhibitor was also shown to suppress gouty arthritis in mice (Xue et al., 2021). However, another recent study showed that NLRP3 inhibitor did not significantly suppress MSU crystal-induced necrosis in macrophages (Zhong et al., 2021). This discrepancy indicates that more experiments need to be performed to clearly elucidate the role of NLRP3 in MSU crystal-induced gouty arthritis. On the other hand, considering that our previous study showed that the active flavonoids could interact with TLR4 by molecular docking (Zhu et al., 2021), we speculate that the active flavonoids might block TLR4 in RAW 264.7 cells. If Raw 264.7 cells were primed with LPS to activate the TLR4/NF-κB pathway, the active flavonoids may not be able to antagonize TLR4 in the presence of LPS. Therefore, the cells were not primed with LPS in the present study. Of course, it cannot be denied that the inflammation induced by MSU was less than that by LPS plus MSU. Therefore, a higher concentration of the active flavonoids was required to inhibit inflammation in the presence of LPS plus MSU.

**FIGURE 5:**
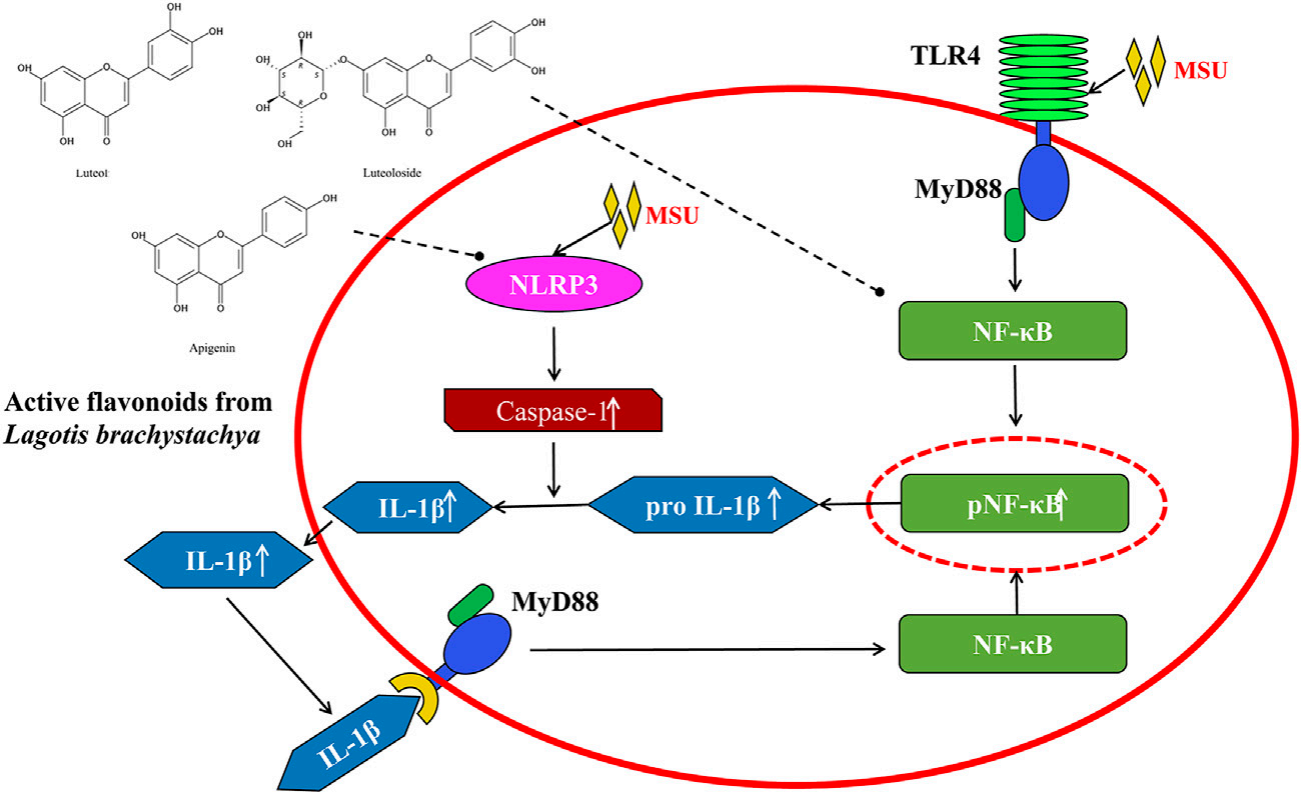
The underlying mechanism involved in the protective effects of the active flavonoids against gouty arthritis.

In conclusion, the present study demonstrated that three active flavonoids (luteolin, luteoloside, and apigenin) from *Lagotis brachystachya* attenuate MSU crystal-induced gouty arthritis *via* inhibiting TLR4/MyD88/NF-κB and NLRP3 expression in the aspect of animal experiment and cell experiment (Figure 5). The specific regulation mechanism may be through the inhibition of inflammatory molecule NF-κB and NLRP3. This inhibition might reduce the levels of proinflammatory factors (TNF-α and IL-1β) and alleviates the inflammatory response in gouty arthritis.

## Data Availability

The original contributions presented in the study are included in the article/Supplementary Material. Further inquiries can be directed to the corresponding authors.
